# The Nursing Supplement

**Published:** 1888-05-05

**Authors:** 


### The Nursing Mirror.

All Contributions for this Supplement should be addressed "The Nursing Mirror," care of The Editor, 38, Old Jewry, E.C.

### En Passant.

A HARD CASE.—Mrs. Webb, the matron of the Lewes Infirmary, has sent in her resignation, after being connected with the institution for 21 years. Mrs. Webb is nearly 74 years of age and is crippled with rheumatism, greatly due to draughts incurred during the late Jubilee alterations, as the roof had to be removed and raised. During all her long years of work Mrs. Webb has been unable to save enough to support her in her old age, having even paid the infirmary washing out of her own small stipend. A movement has very properly been set on foot for presenting her with a testimonial, and any nurses or others who wish to show their sympathy with a sister in distress can send subscriptions to Alfred Addison, 9, Friar's Walk, Lewes.

DIRECTORY FOR NURSES.—Our account of an American Directory of Nurses has excited so much interest that we quote the following further particulars from the first report of the committee: "Up to December 1st, the number of applications by nurses for registration has been 296. Fully registered, 213; approved and awaiting registration, 17; disapproved, 19; stricken from roll for grave faults or defects, 2. Of the registered nurses, there are 26 males and 187 females; 35 graduates of training-schools; 165 non-graduates; manipulators, male and female, 11; cuppers and leechers, 3. The number of calls for nurses has been 342 during the seven months, an average of about 50 a month. We have furnished as many as 8 in a day, and 5 to a single family. We have not, however, a sufficient number of nurses, and especially of trained nurses. Those we have are kept constantly employed, and we need more to supply the demand for the best class of nurses. Boston, with half our population, has 485—double our number of nurses." So successful have the Philadelphia and Boston directories proved, that Washington, Cincinnati, Toronto, San Francisco, and other cities have established, or are establishing, similar institutions.

COTTAGE NURSES.—On Friday last a meeting was held at Reigate, in the Public Hall, to form an association for providing nurses for the sick poor. The nurses are to be women of the humbler class, used to keeping cottages in order, and only partially trained. The nurses are not to give their services free, but labourers are to be encouraged to subscribe 2*s.* annually to the Association, which will entitle them to the nurses' services at 2*s.* a-week when necessary. Artisans, railway porters, &c., are to pay 3*s.* a-week, and farmers and tradespeople 5*s.* a-week. The scheme is not a new one, for a similar association has been at work at Ockley for five years, and has met with great success. It is very hard for the trained city nurse to understand how incapable she is of reaching our country labourers, and how she must here stand aside in favour of her humbler sister, who is known in the locality, and who knows country ways. A nurse is of little use in an isolated cottage who cannot keep the house clean and "do" for the family. She is bound to live as one of themselves and to share their ways; therefore, it is far better she should be accustomed to such things from her youth, and merely have such knowledge of sickness added to her general capacities as the Society can afford to give her. The cases these nurses attend are generally confinements, infectious cases, and certain cases of lung disease, all of which are inadmissible to the cottage hospital. The Society hopes to give all its nurses a certain training in the City of London Lying-in Hospital, for which they will pay five guineas for each nurse pupil.

THE PAY OF PRIVATE NURSES.—We understand that there are some hospitals and institutions which give their nurses a percentage on their earnings, over and above a fixed salary. This seems a very fair arrangement, and one which should cause the nurses to betray more eagerness and interest in procuring cases.

METROPOLITAN NURSING ASSOCIATION.—A concert was given at Grosvenor House on April 1st, in aid of the funds of the South London District Nursing Association, which is a branch of the Metropolitan and National. The performers were the Royal Amateur Orchestral Society, of which the Duke of Edinburgh is president.

CONVALESCENT HOMES.—The busy time is approaching for the Convalescent Homes, and nurses who are tired of the exciting but exhausting work of a hospital can now seek quieter quarters. One small home at Herne Bay is called "The Bird's Nest," and very appropriately opens its doors on May 1st. At Seaford Miss Dowling reigns as matron in place of Miss Eames, and though she has only undertaken the duties temporarily for the summer, there is every hope that she will find them so pleasant as to accept the post permanently. There are only about fifty patients in the Seaford Home at present, but this number will probably be doubled as the season comes on.

THE QUEEN'S NURSING FUND.—On April 20th a meeting was held of the General Committee of the Women's Jubilee Offering at Grosvenor House. The trustees transferred £70,000 to the Nursing Fund, set aside £10,000 for a statue of the Prince Consort, and requested a sub-committee to use the rest in purchasing an article of personal adornment for the Queen. Nurses will be very much interested in watching how this sum is laid out in the interests of the sick poor, and how far they themselves will benefit by the establishment of a good training school for district nursing.

FEMALE SKULLS.—It is said that some years ago, when Miss Frances Power Cobbe was going over the College of Surgeons, she was surprised to find no good specimens of women's skulls. The professor explained to her that only paupers' bodies came to them for dissection, and that they never saw high cranial development among that class. Miss Cobbe immediately offered her own skull to the College when she had done with it, but has lately withdrawn the offer. The College of Surgeons, therefore, is deprived of its promised specimen of a good female skull.

POULTICES.—A correspondent suggests to us that we should write a treatise on poultices as supplied by probationers, and promises we should not find it a short or monotonous subject. It should include the scalding poultice and the damp tepid poultice, the huge heavy poultice and the thin sloppy poultice. Room should also be found to describe the lumpy, sticky and chilly varieties, all of which are to be found daily in hospital life, and all of which are equally unpleasant to the patient. Apparently the perfect poultice is as rare as the perfect woman.

WORK AND LEISURE.—We have received some copies of *Work and Leisure*, a magazine devoted to the interests of women. An account of four months in a cottage hospital is now appearing in it, and there are constant references to nursing work. The inquiry department seems to have quite a number of nurses in want of situations on its books.

### The National Pension Fund for Nurses.

All Communications should be addressed The Editor, The Lodge, Porchester Square, W.

### THE CASE OF THE OLDER NURSES.

I must confess to a certain feeling of disappointment at the tables drawn up for the National Pension Fund, and I fear that I shall not be alone in this feeling. The tables are very well for young nurses, but will, I am afraid, be found quite prohibitory for those over 35 years of age. It seems entirely to have escaped the notice of the compilers that a nurse's salary does not increase with her age, but on the contrary, after a certain age tends rather to diminish. Few institutions of any kind will take on a nurse who is over 40. At any rate, supposing a nurse is at 35 years old earning £30 per annum, her payment to the fund to ensure the minimum sick allowance and £15 pension at 50 would be £13 1*s*. per annum, leaving her only £16 19*s*. per annum for clothes, cost of annual holiday, and other etceras. How could she pay this sum? And most nurses would wish to begin the pension at 50 I think, as the years from 50 to 55 are those in which a woman is least able, as a rule, to work. The whole scale published in the special paper on the advantages of the fund is based upon the age of 25, and £25 per annum as the average salary. But nothing is said as to the disadvantages that older women will have to contend with. I have spoken to several nurses between 35 and 40 years of age, and they all say the money is more than they can afford. In the first instance it was proposed that the payment should be one eighth of a nurse's salary, but the scale is very different to this. It seems to me that the payment should be equal in all cases, taking £25 as the standpoint for salary, so that the greater number of years for which the younger nurses pay in should counterbalance the shorter periods of the older ones. Or, as it is so necessary that a certain number of nurses should join within the first two years, could not a bye-rule be made that all nurses who join during the first year should pay an equal premium, and let the higher scale prevail after then? All the older nurses who did not join within the first year would deserve to reap the consequences. A further question has been suggested to me. You state in this week's Hospital that probably contributions will not commence till one thousand nurses have sent in their applications. Now it may be six months or longer before this number is reached. How will this affect the age and payments of those who send in their papers at once? I have in my mind the case of a nurse who is 39, but who in two months time will be 40. If she sends in her name now will she pay at the rate for 39 years of age, even though she may have turned 40 before the contributions commence? Or will she (which would be rather hard lines) have to pay at the rate for her actual age at the time the contributions are called for? If so, she may as well wait the two months before sending in. Also, will a nurse's payments be reckoned as due from the date of her application, or only from the time the call is made? If the former, and there be long delay, many a nurse might have to pay two or more quarters at once, which they would not like. These points need clearing up before the scheme can be really workable. My own feeling is that contributions should begin at once. People would then realise that the fund is really afloat, and this knowledge would encourage others to join without delay. Kindly insert this letter in the Nursing Supplement.

Elinor Agnes Bedingfeld, Superior,

The Belgravia Institute for Trained Nurses.

[It was, of course, inevitable that at the outset any pension fund founded upon sound business principles must fix rates, which would be higher than many nurses over 35 years of age could afford. To meet such cases two courses have been taken. (1.) Table V. has been prepared, which will enable a nurse to cease all payments to the Fund at 50, and to receive two-thirds pension, *i.e.*, £10 at that age, and the remaining third, *i.e.*, £5, as an addition to her resources at 60. (2.) Every nurse of 35 years and upwards is invited to write to the Secretary of the Fund, 38, Old Jewry, E.C., for a special form, providing she is willing to contribute one-eighth of her earnings to the Fund. Very great sympathy is felt for the older nurses, and everything possible will be done to help those of them who apply at once for a special form, and frankly state their case for the private information of the Council. If this step is taken immediately by the older nurses some such solution as Mrs. Bedingfeld proposes may be found possible. We have ascertained that contributions may begin at once, and that some nurses have already paid their money. If 1,000 nurses do not apply before January 1st, 1890, although this now seems improbable from the fact that nurses are joining the Fund in large numbers every day, the donors of the £25,000 will have to determine what course shall be adopted. If everyone will follow Mrs. Bedingfeld's example and frankly state their difficulties and views, the numbers joining the Fund will soon amount to hundreds instead of tens, as the minimum number of 1,000 applications contemplated.—Ed. *T. H.*]

### THE NATIONAL PENSION FUND AND THE BRITISH NURSES' ASSOCIATION.

My attention has been called to a statement in The Hospital to the effect that the *Nursing Record* must be the organ of the British Nurses' Association, because some of the articles which appeared in the paper were by members of the British Nurses' Association. On the same grounds it would be possible to accuse the *Nineteenth Century* of being the organ of Home Rule if it published an article on "Women's Dress," by Mr. Parnell. On this slender assumption, however, The Hospital proceeds to argue that the British Nurses' Association is trying to wreck the Pension Fund for Nurses. To my certain knowledge this curious deduction is absolutely incorrect; many of the members of the Association have, on the contrary, assured me that they were anxious to join the Fund, and would have done so could they have afforded its terms. The signed articles to which The Hospital refers as a proof of the connexion of the British Nurses' Association with the *Nursing Record*, and the animus of its members to the Pension Fund, are "The Legal Status of Nurses," "Private Nursing," and "Reminiscences of a Small-pox Epidemic." What *have* these subjects to do either with the Pension Fund or (directly) with the British Nurses' Association? It is ridiculous to suppose that because we happen to belong to the British Nurses' Association we should be debarred from contributing papers on nursing subjects to a respectable paper intended for nurses. I sign my name to any papers I may contribute, as an individual, and *not* as a representative of the British Nurses' Association, whose views I do not by any means necessarily embody when I state my own personal opinions. Trusting that in common justice you will insert this in your next number,—I am, yours faithfully,

M. Mollett, Matron.

Chelsea Infirmary, Cale Street, S.W.

[We are glad to publish Miss Mollett's letter, and to be assured on her authority that many of the members of the British Nurses' Association are not unwilling to join the National Pension Fund. If the nurses Miss Mollett refers to will write to the Secretary for a special form they will find they can afford these terms, which are an eighth of the nurse's earnings only.—Ed. *T. H.*]

### APPLICANTS' DIFFICULTIES.

[All questions should be sent to the Secretary, the National Pension Fund, 38, Old Jewry, E.C.]

Questions asked by Applicants and Answers Sent.

*(Continued from p. x.)*

22. Would the fact of entering for an annuity at 50 or 55 (Tables I. and II.) exclude from the benefits of the bonus fund?

The rates for pensions are given in the prospectus—Tables I.

and II. Policies under both these tables will share in the bonus fund.

23. What pension would the payment of £220 secure for a nurse 63 next birthday?

£18 8*s.* per annum for life.

24. A nurse of 56 writes: "I would like to thank the Deputy-Chairman for his kindness. I am sure he is a good friend to hospital nurses. I think more nurses would enter the Fund if the pension could be arranged to commence at the end of 21 years of hospital life. Very few nurses will need a pension after 35 years' hospital life."

The tables in the prospectus are arranged to meet this point. Thus a nurse who is 29 years of age may secure a minimum pension of £15 a-year for life by 21 payments of (a) £8 0*s.* 8*d.* per annum, if the contributions are not returnable; or (b) £9 per annum if she desires to have the right to withdraw them in whole or in part at any time. A nurse of 34 could obtain the same pension by 21 payments of £6 19*s.* 8*d.* in the first case and £8 4*s.* in the second. If these rates are considered too high, a nurse aged 29, by 21 payments of £8 1*s.* 4*d.* can secure a minimum pension of £10 for life, with a minimum increase of £5 more per annum for life at the expiration of another ten years without further payment of any kind. These examples might be indefinitely multiplied.

25. Miss Kate Laczkovie asks (1) if asylum nurses are eligible to enter for sick pay? (2) whether the premiums to be paid will be governed by the nurse's age at the date of her application or at the time when 1,000 applications have been received?

(1) Yes. (2) According to her age at the time of her application. Those nurses who apply at once will therefore get the fullest benefits.

### Everybody's Opinion.

### THE ROUGH SIDE OF HOSPITAL LIFE.

E. L. writes:—"I wish you would speak strongly about the rough side of hospital life for nurses, the bad living and short time allowed for meals. Also about girls taking up the work for a living, who have no taste or love for the thing, and the horrible want of charity and religion they therefore show. On all these points I have strong opinions, but cannot put them into form."

### A PROBATIONER'S PROTEST.

IN this week's issue of THE HOSPITAL, in the Supplement, under the heading of "The Pay of Private Nurses," I read the following: "A probationer gets £10 or £12 during her first year for being of very little use." Now, with your permission, I should like to correct this mistaken idea. I myself have been a probationer in two hospitals, in one for six months, in the other for twelve months, and I can honestly certify that the work of nursing in both these institutions was carried on mainly by the probationers. The "staff" nurses were so many overseers or taskmistresses, and usually got all the praise and honour for the successful and continuous hard work of the probationers. Under these circumstances I think you will agree with me that it is hardly fair to say that the latter are of little use. "Honour to whom honour is due!" NURSE V——.

Edinburgh.

### Appointments.

MISS FANNY M. GRAVELY has been appointed matron of Lewes Infirmary. Miss Gravely is the daughter of a medical man and niece of Dr. Gravely of Newich. She was trained at the Sussex County Hospital, and had charge there of one of the accident wards. There were thirty-five applicants for the place, most of them ladies, though the post is that of working matron.

Two Miss Calverts, who have been trained at Guy's Hospital, have been appointed sisters at St. Saviour's Infirmary.

MISS GODOLPHIN-OSBORNE has been appointed matron of the Home for Incurable Diseases, Leamington. She was trained at the Winchester Hospital, and lately held the post of night superintendent at St. Bartholomew's.

### Anecdotes of Personal Adventure.

OUR visiting physician was getting on in life, very absent minded, and always in a great hurry; hence the following occurrence: He was going round the ward one day, and came to a bed where the patient had just died. It was always a screen case, so before I could stop him he popped his head round the screen, and remarked: "How are you to-day, my man? Better? That's right. Go on just the same."

COMING home from visiting a case one evening, I was accosted by a couple of women, who asked me if I would just step along a few streets to see a man who was desperate bad. I told them they must make proper application to the vicar for my services, but they insisted that the man was dying and nobody doing anything for him. Finally I consented to follow them, and they led me some way out of my own district, into a part of the town I knew little of. "Now nurse," said one woman suddenly, "you must promise not to speak of what you see." I tried to refuse to promise, but the women grew threatening in their tone, all the while dragging me forward through unknown streets. Presently we entered a house in a narrow court and descended into a kind of cellar. It was horribly dark and stuffy and untidy, and on a small wooden bed lay a man groaning loudly. A candle was lit, and by its aid I found that my patient was severely cut from head to foot; indeed, there could be no doubt that he had fallen through some skylight or window. I dressed the great gashes as best I could, the people refusing to send for a doctor. They tried to persuade me to say I would come again secretly, the next day, but I absolutely refused. I was decidedly relieved when the two women had conducted me back into familiar ways once more, and after much hesitation and consultation with the vicar, I communicated my adventure to the police. They never, however, succeeded in tracing my patient.—*L. M.*

### Notes and Queries.

TO CORRESPONDENTS.—1. Questions may be written on post-cards. 2. Advertisements in disguise are inadmissible. 3. In answering a query please quote the number. 4. If a private answer is desired, a stamped addressed envelope must be forwarded.

(11) Would you kindly inquire through your valuable paper if there is any hospital in which a young lady of 21 could be received for training free of charge.—" Probationer," Blackheath.

### Vacancies.

The following vacancies are announced:—

**Matrons.**

Charlton Cottage Hospital.
Hangleton Hospital, Hove; £50.

**Nurses.**

Worcester General Infirmary; £27

Hospital for Chest Diseases, London; £22

London Fever Hospital, Night Superintendent; £30.

London Ophthalmic Hospital; £22

Victoria Home, Bournemouth.

Windsor Infirmary, £20.

District Infirmary, Dewsbury; £20.

Edinburgh Poorhouse Superintendent, £30; Nurse, £25.

Worthing Infirmary; £20.

Children's Hospital, Birmingham. Holiday Nurse.

Children's Hospital, Sheffield.

Birkdale Home, Southport.

Miss Boor's Home, Nottingham.

Dewsbury Infirmary; £20.

Workhouse Infirmary Nursing Association; several.

Victoria Home, Bournemouth; several.

Charlton Cottage Hospital.

**Probationers.**

Lowestoft Hospital.

Garrison Female Hospital, Woolwich; £19.

District Infirmary, Dewsbury.

Bristol Hospital for Sick Children and Women; several.

**District Nurses.**

Dublin.
Reigate.

### Exchange and Mart.

UNDER this heading we propose to insert purely Exchange advertisements, at a charge of sixpence for eighteen words, or three insertions for one shilling It must be distinctly understood that ordinary advertisements will not be inserted here.

### Lectures.

A lecture will be given at the Midwives' Institute and Trained Nurses' Club, 15, Buckingham Street, Strand, on Friday evening, May 11th, at a quarter to eight, subject "Burns and Scalds." The Secretary has a few tickets, which can be obtained at the Club for Nurses and others who are non-members, price sixpence each.

May 4th, Parkes' Museum, at eight p.m., Mr. Shirley F. Murphy on "Infectious Diseases and Methods of Disinfection."

### Examination Questions.

The following are specimens of questions set to probationers in a Provincial Hospital.

Write down in order the things required:—(1) An antiseptic dressing. (2) Paracentesis. (3) For opening an abscess. (4) For a plaster of Paris jacket. (5) For pulling up a fractured thigh. (6) For putting a case of strangulated hernia to bed.

The following are questions asked last year in a Scotch Training School:—

(1) Describe the circulation of the blood. (2) What chief points would you notice in nursing a case of tracheotomy? (3) Write down apothecaries' weight and liquid measure. What is the normal temperature of the body, beats of the pulse per minute, respirations per minute? (5) What are the rules to be observed in a case of enteric fever? (6) How would you prepare an operating table in a private house?

### MR. BURDETT'S BOOKS ON HOSPITALS.

"Mr. Henry C. Burdett, whose interest in Hospitals is well known, is widely recognised as the chief authority on the subject."—*The Lancet*, July 25th, 1885.

**HOSPITALS AND THE STATE: HOSPITAL MANAGEMENT, AND HOSPITAL NURSING.** With Statistics of the Chief Medical Institutions in Great Britain and Ireland. [Cloth, 2s. 6d.

**COTTAGE HOSPITALS: GENERAL, FEVER, AND CONVALESCENT.** Their Progress, Management, and Work. With an Alphabetical List of every Cottage Hospital at present opened, and Chapters on Mortuaries, Convalescent Cottages, and the Relative Mortality in Large and Small Hospitals. The book contains the ground-plan and elevation of the best constructed British Hospitals and Medical Institutions having fifty beds and under, and also a Portrait of Albert Napper, Esq., the Founder of Cottage Hospitals. Second Edition. 600 pp. [Cloth, 14s.

**PAY HOSPITALS AND PAYING WARDS THROUGHOUT THE WORLD:** Facts in support of a Re-arrangement of the English System of Medical Relief. 200 pp. [Demy 8vo., 6s.

**SELECTED EXTRACTS FROM REVIEWS.**

*British Medical Journal.*—"Mr. Burdett is well known to the medical profession as an advocate of provident dispensaries and hospitals, and as the originator of the Home Hospitals Association. All who are interested in these subjects will be gratified by this volume. In it the author deals with the whole question of pay hospitals and dispensaries in a large and comprehensive spirit. We commend the book to all who are interested in the improvement of medical relief—and which of us is not? It is clearly and pleasantly written, it is full of facts, and it contains many valuable suggestions. This work cannot fail to stimulate the reforming movements which have been gathering strength in this country during the last few years.'

*New York Medical Journal.*—"Mr. Burdett has devoted many years to the study of hospital administration and management, and his book and plans are well worth perusal."

*The American Practitioner.*—"Mr. Burdett displays and discusses the whole scheme of hospital accommodation with a comprehensive understanding of its nature and extent, and he does it in fulness without prolixity, and in a clear catholic spirit with perspicacity. The book is a stepping-stone, a valuable contribution in the way of introduction to a review and candid reconsideration of the whole subject of legal and organised charity—a theme which much demands consideration and re-adjustment in the whole civilised world. A good and timely book, and suggestive.'

London: J. and A. CHURCHILL, 11, New Burlington Street, W.

